# Pulmonic Valve Endocarditis with Pulmonary Artery Endarteritis in a Young Man with Congenital Ventral Septal Defect

**Published:** 2010

**Authors:** Afsoon Fazlinezhad, Azadeh Fallah, Jamil Esfahanizadeh

**Affiliations:** 1 Associate Professor of Cardiology, Department of Echocardiography, Ghaem Hospital, Mashhad University of Medical Sciences, Mashhad, Iran; 2 Resident of Cardiology, Department of Cardiology, Ghaem Hospital, Mashhad University of Medical Sciences, Mashhad, Iran; 3 Assistant Professor of Cardiac Surgery, Department of Cardiac Surgery, Ghaem Hospital, Mashhad University of Medical Sciences, Mashhad, Iran

**Keywords:** Infective endocarditis, Pulmonic valve, Vegetation, Endartritis

## Abstract

**BACKGROUND:**

Isolated pulmonic valve endocarditis is a rare condition. The clinical and laboratory finding are not specific and experiences about that are limited. Most cases of that occur in children with congenital heart disease or in intravenous drug abusers and the main predisposing factor in adults is intravenous drug abuse. The most common pathogens are staphylococcus aurous and coagulase negative staphylococcus.

**CASE REPORT:**

In this case report we present a 27 years old man with chronic fever (4 months) and a history of congenital ventral septal defect (VSD). Echocardiography revealed the pulmonic valve and pulmonary artery vegetations. He referred for surgery after 3 weeks of intravenous antibiotic therapy.

**CONCLUSION:**

Careful evaluation of pulmonic valve in echocardiography should be done, when ever vegetation is not detected in other valves, and clinical suspicion for infective endocarditis is high.

## Case Report

A 27 years old man with fever, rigors, night sweating, severe weight loss (about 15 Kg) from 4 months ago was admitted to our hospital. On admission, he was ill, febrile and suffered from pleuretic chest pain. He was in good health until 4 months ago. The patient didn't have history of IV drug abuse.

**Figure 1:**
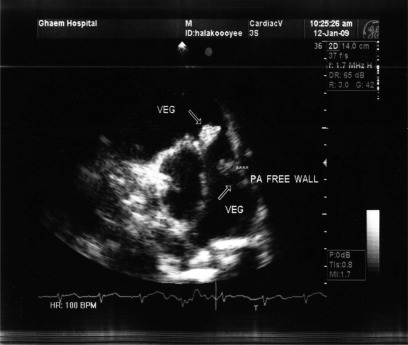
Parasternal short axis view revealed two vegetations on pulmonic valve and PA free wall

**Figure 2:**
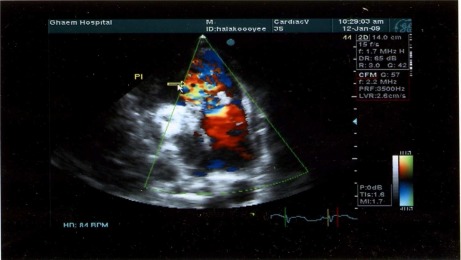
Parasternal short axis color Doppler imaging revealed severe free pulmonic insufficiency

He had an episode of sub massive hemoptesia a week before admission. On physical examination, he appeared febrile, anemic, and toxic. Blood pressure was 110/70 mmHg, heart rate was 105 bpm and regular. Jugular veins were distended. On auscultation, there was 4/6 grade to-and-fro murmur at pulmonary area without radiation and another 5/6 grade holosystolic murmur in left sternal border. Multiple sub conjunctive petechia, osler nodes on pulm of the left upper limb and clubbing was also seen. Electrocardiography showed sinus tachycardia with right axis deviation. Chest x-ray showed cardiomegaly with multiple patchy infiltrations in both lung parenchyma. Transthoracic echocardiography showed large highly mobile bulky vegetation on pulmonic valve with valve destruction and severe free pulmonic insufficiency (Figure 1, 2).

Another mobile vegetation was also seen on pulmonary artery free wall with significant thickening and inflammation of endothelium (Figure 1). There was also small sub aortic ventral septal defect (VSD) (defect = 5 mm) and secondary aortic valve prolapse and mild aortic insufficiency. No vegetation on other valves was seen.

Laboratory data indicated anemia (Hg = 7.5 gr/dl, PMN = 85%), ESR was 113, and CRP was positive.

Axial thorax CT scan showed multiple diffused nodules especially in basal portion.

**Figure 3:**
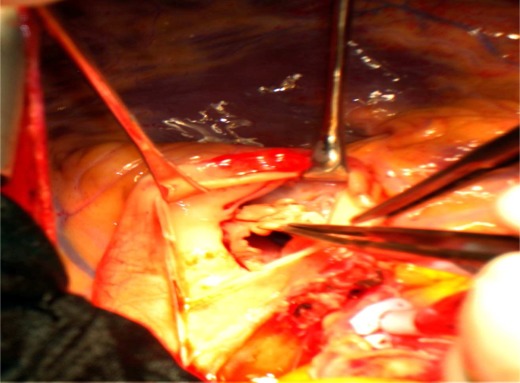
Macroscopic surgical view revealed vegetation on pulmonic valve

Respect to Duke Criteria, infective endocarditis was confirmed and full dose antibiotic therapy was started. Due to recurrent pulmonic septic emboli, the patient referred to surgical department for surgery, pulmonic valve replacement with bioprosthesis, removal of pulmonary artery vegetation and VSD closure was performed (Figure 3) and patient was discharged 2 weeks after surgery without any complication.

## Discussion

Tricuspid valve is the most common right sided valve involved in infective endocarditis, either alone or in conjunction with pulmonary valve.^1^ Isolated pulmonic valve endocarditis is an uncommon clinical entity. The clinical and laboratory findings are not specific and the accumulated experience about it is limited.^2^ It shares epidemiologic, clinical, radiologic and microbiologic feature with tricuspid valve infective endocarditis.^3^ The main predisposing factors for pulmonic valve infective endocarditis in adults are intravenous drug abuse in 30% of cases, central venus catheters in 14%, and alcoholism in 11%.^4^, ^5^ Isolated pulmonic valve infective endocarditis has also been identified in patients undergoing chronic hemodialysis, liver transplantation and celiac disease.^6^–^9^ Many cases are unsuspected and become evident after echocardiography or autopsy.^7^ The most common pathogens that are reported in cases of pulmonic valve infective endocarditis, are staphylococcus aurous and coagulase negative staphylococcus.^10^ Septic pulmonary emboli occur in up to 75% of patients.^11^ Reviews of the published clinical experience indicate that the role of surgery in isolated pulmonary valve infective endocarditis is unclear.^11^

## Conclusion

In this report, we presented a case with pulmonic valve endocarditis and pulmonary artery endarteritis in a young man with small congenital VSD, which was undiagnosed for 4 months. It is important and should be kept in mind that infective endocarditis may have confusing clinical features. Careful evaluation of pulmonic valve in echocardiography should be done, when ever vegetation is not detected in other valves, and clinical suspicion for infective endocarditis is high.
